# Aggravation of ovalbumin-induced murine asthma by co-exposure to desert-dust and organic chemicals: an animal model study

**DOI:** 10.1186/1476-069X-13-83

**Published:** 2014-10-18

**Authors:** Yahao Ren, Takamichi Ichinose, Miao He, Keiichi Arashidani, Yasuhiro Yoshida, Seiichi Yoshida, Masataka Nishikawa, Hirohisa Takano, Guifan Sun, Takayuki Shibamoto

**Affiliations:** Department of Nutritional and Food Hygiene, College of Public Health, China Medical University, Shenyang, China; Department of Health Sciences, Oita University of Nursing and Health Sciences, Oita, Japan; Environment and Chronic Non-communicable Disease Research Center, School of Public Health, College of Public Health, China Medical University, 11001 Shenyang, China; Department of Immunology and Parasitology, School of Medicine, University of Occupational and Environmental Health, 807-8555 Fukuoka, Japan; Environmental Chemistry Division, National Institute for Environmental Studies, 305-8506 Ibaraki, Japan; Environmental Health Division, Department of Environmental Engineering, Graduate School of Engineering, Kyoto University, 615-8530 Kyoto, Japan; Department of Environmental Toxicology, University of California, Davis, CA 95616 USA

**Keywords:** Tar, Polycyclic aromatic hydrocarbons, Asian sand dust, Ovalbumin, Lung eosinophilia, Cytokine and chemokine

## Abstract

**Background:**

The organic chemicals present in Asian sand dust (ASD) might contribute to the aggravation of lung eosinophila. Therefore, the aggravating effects of the Tar fraction from ASD on ovalbumin (OVA)-induced lung eosinophilia were investigated.

**Methods:**

The Tar fraction was extracted from ASD collected from the atmosphere in Fukuoka, Japan. ASD collected from the Gobi desert was heated at 360°C to inactivate toxic organic substances (H-ASD). ICR mice were instilled intratracheally with 12 different test samples prepared with Tar (1 μg and 5 μg), H-ASD, and OVA in a normal saline solution containing 0.02% Tween 80. The lung pathology, cytological profiles in the bronchoalveolar lavage fluid (BALF), inflammatory cytokines/chemokines in BALF and OVA-specific immunoglobulin in serum were investigated.

**Results:**

Several kinds of polycyclic aromatic hydrocarbons (PAHs) were detected in the Tar sample. H-ASD + Tar 5 μg induced slight neutrophilic lung inflammation. In the presence of OVA, Tar 5 μg increased the level of eosinophils slightly and induced trace levels of Th2 cytokines IL-5 and IL-13 in BALF. Also mild to moderate goblet cell proliferation and mild infiltration of eosinophils in the submucosa of airway were observed. These pathological changes caused by H-ASD + OVA were relatively small. However, in the presence of OVA and H-ASD, Tar, at as low a level as 1 μg, induced severe eosinophil infiltration and proliferation of goblet cells in the airways and significantly increased Th2 cytokines IL-5 and IL-13 in BALF. The mixture showed an adjuvant effect on OVA-specific IgG1 production.

**Conclusions:**

These results indicate that H-ASD with even low levels of Tar exacerbates OVA-induced lung eosinophilia via increases of Th2-mediated cytokines. These results suggest that ASD-bound PAHs might contribute to the aggravation of lung eosinophila.

## Background

Ambient particulate matter (PM)_2.5_ (particle sizes less than 2.5 μm) and PM_10_ (particle sizes of 2.5 μm ~10 μm) are positively related to an exacerbation of respiratory diseases like asthma and chronic bronchitis [1, 2]. The health issue by desert-dust (—well known as PM_10_—) has arisen all over the world [3–6]. Asian sand dust (ASD) storms arise annually from the Gobi Desert, the Taklimakan desert, and loess areas of interior China during the spring season and/or sometimes during the autumn season every year. When a large scale sandstorm occurs from these deserts, ASD aerosol spreads over large areas, including East China, the Korean Peninsula and Japan as well as crossing the North Pacific to the United States [7–9].

Some reports suggest that ASD events are associated with increased respiratory symptoms in both adults and children with asthma [6, 10, 11]. Similar to these epidemiological investigations, our previous studies showed an aggravating effect of ASD on ovalbumin (OVA)-induced lung eosinophilia in a murine model of asthma. On the other hand, if ASD is heated (H-ASD) at 360°C to inactivate microbial materials and chemicals, it caused fewer effects [12, 13]. On the basis of these results, we hypothesized that the organic substances and chemicals adhering to ASD contribute to the aggravation of lung eosinophila. Recently, we showed that ASD contaminated with a trace level of bacterial lipopolysaccharide (LPS) aggravated murine lung eosinophilia [14].

By-products, such as organic chemical, sulfates (SO_4_^2−^) and nitrates (NO_3_^−^), formed from the combustion of coal and other fossil fuels in industrialized Eastern Asia adhere to ASD during long-range transportation of the dust [15–17]. PM2.5, such as diesel exhaust particles (DEP), is present in the Tar fraction, which contains polycyclic aromatic hydrocarbons (PAHs). Previous studies have reported that Tar fraction extracted from DEP containing PAHs exacerbated OVA-induced experimental asthma [18, 19]. Therefore, it should be ascertained whether Tar fractions extracted from ASD are related to the aggravation of lung eosinophilia by ASD.

In the present study, the exacerbating effects of Tar and/or H-ASD on OVA- induced lung eosinophilia were investigated using ICR mice.

## Methods

### Animals

Specific pathogen-free male ICR mice (6 weeks of age) were obtained from Charles River Japan, Inc. (Kanagawa, Japan). The mice were observed for 1 week to measure body weight and to eliminate those who showed signs of infection. The mice used in the study were 7 weeks of age. CE-2 commercial diet (CLEA Japan, Tokyo, Japan) and water were given ad libitum. The mice were housed in plastic cages lined with soft wood chips. The cages were placed in a conventional room, which was air conditioned at 23°C with a light/dark (12 h/12 h) cycle, and humidity ranging from 55 to 70%. The study adhered to the US National Institutes of Health guidelines for the use of experimental animals. The animal care method was also approved by the animal care and use committee at Oita University of Nursing and Health Sciences in Oita, Japan.

### Sample preparations and analysis of Tar components

ASD collected from surface soils in the Gobi desert was purified for use as the standard in the present study. The size distribution peak was observed at 3.9 μm. The chemical elements in ASD were as reported previously: 51.6% SiO_2_, 14.3% Al_2_O_3_, 5.5% Fe_2_O_3_, 1.3% Na_2_O, 9.6% CaCO_3_, 0.6% CaO, 2.5% MgO, 0.7% TiO_2_ and 2.6% K_2_O. And, as in the previous study, 11.3% of total oxides were lost at ig [14, 20]. PAHs were not detected in the standard ASD. A portion of the standard ASD was heated at 360°C for 30 min in an electric heater to inactivate toxic materials (sulfate, nitrate, microorganism, etc.). These samples are termed H-ASD in the present study.

The ASD that was the focus of this study was collected from the atmosphere at the University of Occupational and Environmental Health, Kitakyushu, Fukuoka, Japan on November 13–15, 2010—during a massive dust storm event in East Asia—using a high-volume air sampler (Sibata Scientific Technology, Japan) equipped with a quartz-filter (20 × 25 cm, 2500QAT-UP, Tokyo Dylec Corp, Japan). After the ASD was trapped on a quartz filter, the quartz filter (20 × 25 cm, 2500QAT-UP, Tokyo Dylec Corp, Japan) was cut into 1/8 piece. The pieces of cut filter were extracted twice with a 20 mL portion of dichloromethane (Wako Pure Chemicals, Industries Ltd, Japan) for 15 min at 15°C by ultrasonic extraction. The extract was filtered with an NO 5C filter paper and the filtrate allowed to stand in the dark until the solvent was evaporated to dryness to yield solid materials. Tar 13.4 mg (3.35% w/w) was obtained from 400 mg of ASD.

The residual materials were dissolved in 0.5 mL acetonitrile then analyzed for PAHs by a Hitachi Model 600 HPLC (Hitachi, Japan) equipped with a Model L-7485 fluorescence detector (Hitachi, Japan) and a 4.0 mmφ × 250 mm column packed with Wakosil-II 5C 18HG (Waka Pure Chemicals Industry, Ltd., Japan). The mobile phase was acetonitrile/water (80/20, v/v) at 1.5 ml/min.

Identification of PAHs in the sample was conducted by comparison of the HPLC retention time and fluorescence/excitation spectra to those of authentic PAHs according to a previously reported method [21]. Authentic PAHs were obtained from Supelco (Bellefonte, PA, USA), Aldrich Chemical Co., Inc (Milwaukee, WI, USA) and Tridom Chemical Inc (Hauppauge, NY, USA).

### Study protocol

ICR mice (n = 168) were divided into 12 groups (n = 14 per group) and each group was treated with a specific testing sample. The 12 test samples were composed of a 0.1 mL basic saline solution (0.9% NaCl and 0.02% Tween 80) and the various testing materials shown in Table 1.

**Table 1:** **Amounts of Tar, H-ASD and OVA contained in each sample**

Sample	Tar	H-ASD	OVA
Control	0	0	0
Tar 1	1 μg	0	0
Tar 5	5 μg	0	0
H-ASD	0	0.1 mg	0
H-ASD + Tar 1	1 μg	0.1 mg	0
H-ASD + Tar 5	5 μg	0.1 mg	0
OVA	0	0	1 μg
OVA + Tar 1	1 μg	0	1 μg
OVA + Tar 5	5 μg	0	1 μg
OVA + H-ASD	0	0.1 mg	1 μg
OVA + H-ASD + Tar 1	1 μg	0.1 mg	1 μg
OVA + H-ASD + Tar 5	5 μg	0.1 mg	1 μg

The instillation dose of H-ASD was 0.1 mg per mouse [14]. Airborne ASD contains about 1% - 5% tar. Therefore, the instillation doses of Tar were set to 1 μg and 5 μg per 0.1 mg ASD, to be consistent with the proportion of Tar to ASD generally. The mice were intratracheally challenged with each sample 4 times at 2-week intervals. One day after the last intratracheal administration, mice from all groups were euthanized by exsanguination under deep anesthesia by intraperitoneal injection of pentobarbital. Serum was taken by centrifugal separation of blood. The serum samples were stored at −80°C until analysis for OVA-specific immunoglobulin E (IgE) and IgG1 antibodies.

### Bronchoalveolar lavage fluid (BALF)

Eight out of the 14 mice in each group were examined for the free cell contents in BALF using a previously reported method [22, 23]. Briefly, the lungs were lavaged twice with an injection of sterile saline solution (0.8 ml) at 37°C. After the fluids from the two lavages were combined and cooled to 4°C, the resultant solution was centrifuged at 1500 rpm for 10 min. The total cell count of the fresh fluid speimen was determined by a hemocytometer. Differential cell counts were assessed on cytological preparations. Slides were prepared using a Cytospin (Sakura Co., Ltd, Tokyo, Japan) and stained with Diff-Quik (International Reagents Co., Kobe, Japan) to identify the eosinophils with red granules. A total of 300 cells were counted under microscopic examination. The BALF supernatants were stored at −80°C until analysis for cytokines and chemokines.

### Pathological evaluation

The remaining 6 mice in each group were used for pathological examination. The lungs were fixed by a 10% neutral phosphate-buffered formalin solution. After separation of the lobes, 2-mm-thick blocks were taken for paraffin embedding. Embedded blocks were sectioned at a thickness of 3 μm and then stained with hematoxylin and eosin (H & E) to evaluate the degree of infiltration of eosinophils and lymphocytes in the airway from proximal to distal. The sections were stained with periodic acid-Schiff (PAS) to evaluate the degree of proliferation of goblet cells in the bronchial epithelium. A pathological analysis of inflammatory cells and epithelial cells in the airway was performed using a Nikon ECLIPSE light microscope (Nikon Co., Tokyo, Japan). The degree of infiltration of eosinophils and lymphocytes in the airway or proliferation of goblet cells in the bronchial epithelium was graded in blinded fashion as: 0, not present; 1, slight; 2, mild; 3, moderate; 4, moderate to marked; 5, marked. ‘Slight’ was defined as less than 20% of the airway with eosinophilic inflammatory reaction or with goblet cells stained with PAS; ‘mild’ as 21 - 40%; ‘moderate’ as 41 - 60%; ‘moderate to marked’ as 61 - 80%; and marked as more than 80% of the airway [22, 23].

### Quantitation of cytokines and chemokines in BALF

The cytokine and chemokine protein levels were determined by enzyme-linked immunosorbent assays (ELISA). IL-5 and IL-12 were measured using an ELISA kit from Endogen, Inc. (Cambridge, MA, USA). MCP-3 was measured using an ELISA kit from Bender MedSystems Inc. (Burlingame, CA, USA). IL-1β, IL-4, IL-6, IL-13, IFN-γ, KC, TGF-β, Eotaxin, MCP-1, MIP-1α, RANTES were measured using an ELISA kit from R&D Systems Inc. (Minneapolis, MN, USA).

### OVA-specific IgE and IgG1 antibodies

OVA-specific IgE and IgG1 antibodies in serum were measured using the Mouse OVA-IgE ELISA kit and Mouse OVA-IgG1 ELISA kit (Shibayagi Co., Shibukawa, Japan). According to the manufacturer’s protocol, 1U of the anti-OVA IgE is defined as 1.3 ng of the antibody; and 1U of the anti-OVA IgG1 is defined as 160 ng of the antibody. The absorption of 450 nm (sub-wave length, 620 nm) for OVA-specific IgE and IgG1 antibody was measured by a microplate reader (Spectrafluor, Tecan, Salzburg, Austria).

### Statistical analysis

Statistical analysis of pathological evaluation in the airway, cell numbers (macrophages, neutrophils, eosinophils and lymphocytes), cytokines, and chemokine proteins in BALF was conducted using the Tukey Test for Pairwise Comparisons, All the analyses were performed with IBM SPSS Statistics Client21 (AsiaAnalytics, Shanghai, China). Differences were considered significant at p <0.05.

## Results

### Concentration of PAHs in Tar from ASD

PAHs detected in the Tar are shown in descending order of ppm level in Table 2. The concentration of fluoranthene (217 μg/g) was the highest, followed by benzo[e]pyrene (174 μg/g), and indeno[1,2,3-cd]pyrene (122 μg/g). Concentration of the most potent carcinogen, benzo[a]pyrene, was 34.6 μg/g.

**Table 2:** **Concentrations of PAHs in Tar**

PAH	Concentration (μg/g)
Fluoranthene	217
Benzo[e]pyrene	174
Indeno[1,2,3-cd]pyrene	122
Benzo[j]fluoranthene	92.2
Pyrene	73.1
Coronene	64.1
Benz[a]anthracene	60.1
Chrysene	54.8
Benzo[ghi]perylene	52.1
Dibenzo[a,h]anthracene	43.3
Benzo[a]pyrene	34.6
Benzo[k]fluoranthene	27.3
Benzo[b]fluoranthene	20.8
Perylene	9.12

Tar fraction extracted from ASD were analyzed for PAHs by a HPLC equipped with a fluorescence detector and a 4.0mmφ × 250 mm column a packed with Wakosil-II 5C 18HG.

### The cell numbers in BALF from mice treated by OVA, H-ASD and Tar

**Figure 1:**
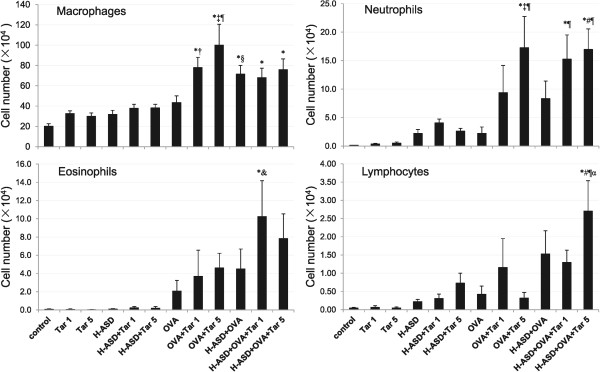
**Cellular profile in bronchoalveolar lavage fluids (BALF).** All values are expressed as mean ± SE. ^*^p < 0.05 vs. control; ^†^p < 0.05 vs. Tar 1; ^‡^p < 0.05 vs. Tar 5; ^§^p < 0.05 vs. H-ASD; ^&^p < 0.05 vs. H-ASD + Tar 1; ^#^p < 0.05 vs. H-ASD + Tar 5; ^¶^p < 0.05 vs. OVA; ^α^p < 0.05 vs. OVA + Tar 5.

Figure 1 shows the cellular profiles in BALF. Tar 1 and Tar 5 increased the number of macrophages by 63% and 50%, respectively compared with the control. The addition of H-ASD to Tar 1 and Tar 5 increased neutrophils and lymphocytes slightly compared with the control. The addition of OVA to Tar 1 and Tar 5 increased eosinophils slightly, and they also make significant increases in macrophages and neutrophils (OVA + Tar 5 group) compared with the non-OVA treated groups. When Tar 1 was added to H-ASD + OVA, eosinophil number increased significantly compared with the OVA alone group.

### Pathological changes in the airways of mice treated by OVA, H-ASD and Tar

**Figure 2:**
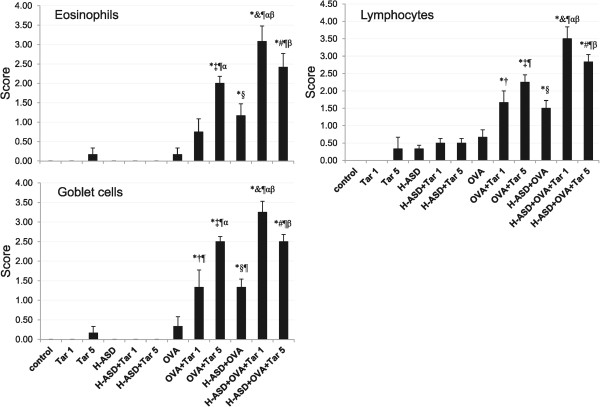
**Evaluation of pathological changes in the murine airway.** The degree of pathological changes in the airway was estimated as: (0) none; (1) slight; (2) mild; (3) moderate; (4) moderate to marked; (5) marked. All values are expressed as mean ± SE (n = 6). Statistical analyses were conducted using the Tukey Test for Pairwise Comparisons. ^*^p < 0.05 vs. control; ^†^p < 0.05 vs. Tar 1; ^‡^p < 0.05 vs. Tar 5; ^§^p < 0.05 vs. H-ASD; ^&^p < 0.05 vs. H-ASD + Tar 1; ^#^p < 0.05 vs. H-ASD + Tar 5; ^¶^p < 0.05 vs. OVA; ^α^p < 0.05 vs. OVA + Tar 1; ^β^p < 0.05 vs. H-ASD + OVA.

Figure 2 shows the pathological changes caused by the testing samples in the murine airway.

When OVA + Tar 5 samples were used for exposures, mild to moderate increases of eosinophils, lymphocytes and goblet cells occurred compared with OVA exposures alone. OVA + H-ASD caused a slight to mild increases of these cells compared with OVA alone. However, when Tar 1 or Tar 5 was added to H-ASD + OVA samples, moderate to marked increase of these cells compared with H-ASD + OVA was observed.

**Figure 3:**
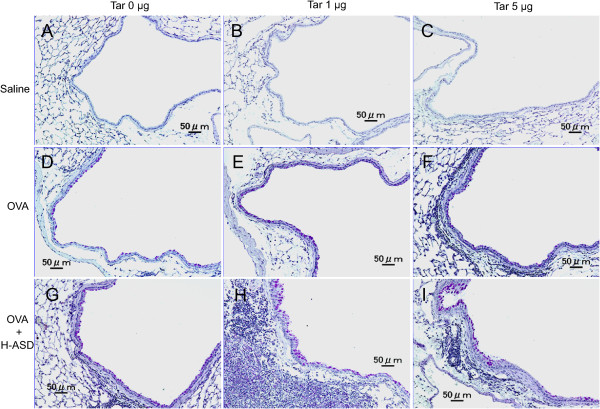
**Effects of test samples on pathological changes in the lungs. (A)** Control, **(B)** Tar 1: no pathologic alterations were seen in the lungs. **(C)** Tar 5: very slight infiltration of inflammatory cells in the airway submucosa. Goblet cells were not seen. **(D)** OVA alone: slight proliferation of goblet cells in the airway epithelium and very slight infiltration of inflammatory cells into the airway submucosa. **(E)** OVA + Tar 1: goblet cell proliferation was somewhat stronger than in the samples treated with OVA alone **(D). (F)** OVA + Tar 5: mild proliferation of goblet cells in the airway epithelium, and infiltration of inflammatory cells into the airway submucosa. **(G)** H-ASD + OVA: mild goblet cell proliferation and mild infiltration of inflammatory cells into the airway submucosa. **(H)** H-ASD + OVA + Tar 1: moderate goblet cell proliferation, severe infiltration of inflammatory cells into the airway submucosa. **(I)** H-ASD + OVA + Tar 5: mild to moderate goblet cell proliferation, moderate infiltration of inflammatory cells into the airway submucosa. **(A–I)** PAS stain; bar = 50 μm.

**Figure 4:**
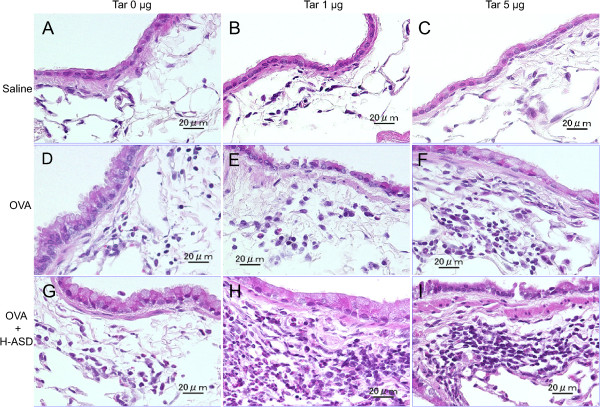
**Effects of test samples on infiltration of inflammatory cells in the airway. (A)** Control, **(B)** Tar 1, **(C)** Tar 5: no pathological changes in lungs treated with saline, Tar 1 and Tar 5. **(D)** OVA alone: slight infiltration of eosinophils, neutrophils and lymphocytes into the airway submucosa. **(E)** OVA + Tar 1, **(F)** OVA + Tar 5, **(G)** H-ASD + OVA: slight to mild infiltration of inflammatory cells into the airway submucosa. **(H)** H-ASD + OVA + Tar 1: marked accumulation of eosinophils, neutrophils and lymphocytes in the airway submucosa. **(I)** H-ASD + OVA + Tar 5: moderate infiltration of these inflammatory cells into the submucosa of airways. **(A–I)** HE stain; bar = 20 μm.

Figures 3 and 4 illustrate the effects of Tar on pathological changes in the lungs. No pathological alterations were found in the lungs of the control and Tar 1 (Figure 4A and B). H-ASD, H-ASD + Tar 1 and H-ASD + Tar 5 caused slight infiltration of inflammatory cells in the submucosa of the airway (data not shown).

Compared with the control group (Figures 3A; 4A), OVA alone caused a very slight proliferation of goblet cells in the airway epithelium (Figure 3D) as well as of eosinophils, neutrophils and lymphocytes in the submucosa of airway (Figure 4D). OVA + Tar 5 caused mild to moderate goblet cell proliferation (Figure 3F) and mild infiltration of eosinophils in the submucosa of airway (Figure 4F) compared with the group of OVA alone (Figure 3D; Figure 4D).

H-ASD + OVA also caused a slight goblet cell proliferation and slight infiltration of eosinophils and lymphocytes in the submucosa of airway (Figures 3G; 4G) compared with OVA alone (Figures 3D, 4D). However, H-ASD + OVA + Tar 1 and H-ASD + OVA + Tar 5 caused moderate goblet cell proliferation in the airway epithelium (Figure 3H, I) and moderate to marked accumulation of eosinophils, neutrophils and lymphocytes in the submucosa of airways (Figure 4H, I) compared with OVA alone (Figures 3D, 4D). These pathological alterations in the H-ASD + OVA + Tar 1 group were more severe than in the H-ASD + OVA + Tar 5 group.

### Protein levels of cytokines and chemokines in BALF from mice treated by OVA, H-ASD and Tar

**Figure 5:**
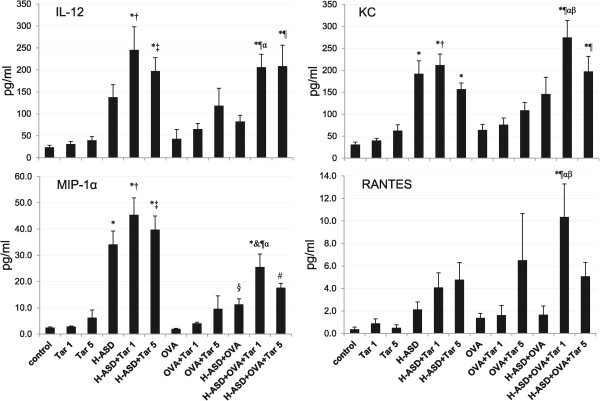
**Expressions of IL-12, KC, MIP-1** **α** **and RANTES in BALF.** All values are expressed as mean ± SE (n =8). ^*^p < 0.05 vs. control; ^†^p < 0.05 vs. Tar 1; ^‡^p < 0.05 vs. Tar 5; ^§^p < 0.05 vs. H-ASD; ^&^p < 0.05 vs. H-ASD + Tar 1; ^#^p < 0.05 vs. H-ASD + Tar 5; p < 0.05 vs. OVA; ^α^p < 0.05 vs. OVA + Tar 1; ^β^p < 0.05 vs. H-ASD + OVA.

Figure 5 shows the levels of IL-12, KC, MIP-1α and RANTES in BALF. Tar 1 and Tar 5 did not increase the proteins examined, whereas H-ASD significantly increased the protein level of MIP-1α and KC, and H-ASD + Tar increased all proteins compared with the control group. The addition of Tar to OVA showed a dose-dependent increase in all proteins. However, when Tar was added to an H-ASD + OVA sample, all proteins did not show a dose-dependent increase; the mice treated by H-ASD + OVA + Tar 1 contained higher protein levels of KC, MIP-1α and RANTES.

**Figure 6:**
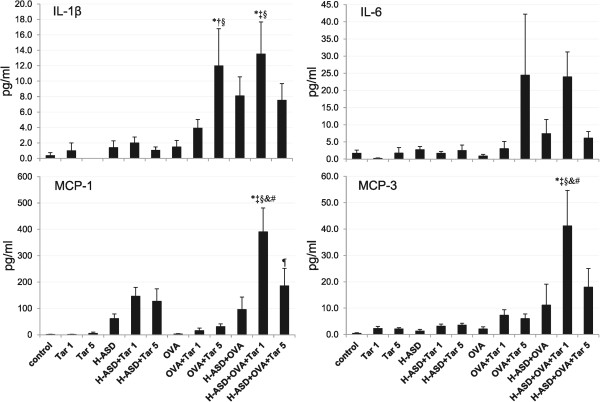
**Expressions of IL-1β, IL-6, MCP-1 and MCP-3 in BALF.** All values are expressed as mean ± SE (n =8). ^*^p < 0.05 vs. control; ^†^p < 0.05 vs. Tar 5; ^‡^p < 0.05 vs. H-ASD + Tar 1; ^§^p < 0.05 vs. OVA; ^&^p < 0.05 vs. OVA + Tar 1; ^#^p < 0.05 vs. H-ASD + OVA; p < 0.05 vs. H-ASD + OVA + Tar 1.

Figure 6 shows the expression of IL-1β, IL-6, MCP-1 and MCP-3. H-ASD and H-ASD + Tar increased the protein level of MCP-1 slightly compared with the control group. The addition of Tar to H-ASD + OVA increased all proteins considerably, and the mice treated by H-ASD + OVA + Tar 1 contained higher levels of all proteins compared with the H-ASD + OVA + Tar 5 group.

**Figure 7:**
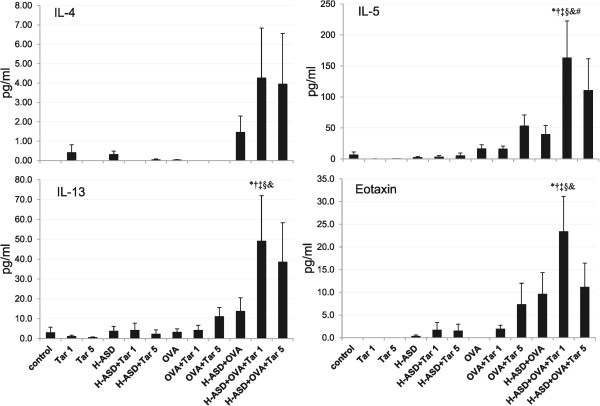
**Expressions of IL-4, IL-5, IL-13 and eotaxin in BALF.** All values are expressed as mean ± SE (n = 8). ^*^p < 0.05 vs. control; ^†^p < 0.05 vs. Tar 1; ^‡^p < 0.05 vs. H-ASD + Tar 1; ^§^p < 0.05 vs. OVA; ^&^p < 0.05 vs. OVA + Tar 1; ^#^p < 0.05 vs. H-ASD + OVA.

Figure 7 shows the expression of IL-4, IL-5, IL-13 and eotaxin in BALF. These proteins are known as allergy associated mediators. There was a slight increase in levels of IL-5 in the samples treated by OVA alone. The addition of Tar 5 to OVA increased IL-13 and eotaxin slightly and caused a further, slight increase of IL-5. Addition of H-ASD to OVA increased all proteins slightly, while the addition of Tar 1 to H-ASD + OVA triggered a remarkable increase in all proteins compared with the other OVA-treated groups.

TGF-β and IFN-γ were not detected in the present study.

### Enhancement of OVA-specific IgG1 by H-ASD and Tar

**Figure 8:**
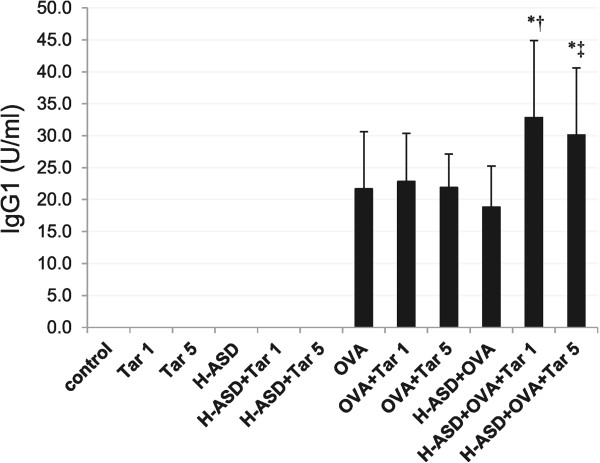
**Effects of test samples on OVA-specific IgG1 production in serum.** According to the manufacturer’s protocol, 1 U of the anti-OVA IgG1 is defined as 160 ng of the antibody. All values are expressed as mean ± SE. ^*^p < 0.05 vs. control; ^†^p < 0.05 vs. H-ASD + Tar 1; ^‡^p < 0.05 vs. H-ASD + Tar 5.

Figure 8 shows the effects of test samples on IgG1 production in serum. IgG1 was not detected in the samples of the control, Tar 1, Tar 5, H-ASD, H-ASD + Tar 1 or H-ASD + Tar 5 samples. The mice treated by H-ASD + OVA + Tar 1 contained the highest level of IgG1, followed by the mice treated by H-ASD + OVA + Tar 5. In the present study, OVA-specific IgE was not detected.

## Discussion

It is possible that desert dust-bound organic chemicals contribute to the aggravation of allergic lung inflammation. The present study demonstrated that Tar adhering to ASD contributed to the aggravating effect on OVA-induced lung eosinophilia. The Tar used in the present study contained high concentration of PAHs, suggesting that the aggravating effect by Tar greatly due to PAHs. However, the effects of pro-inflammatory mediators and lung pathology in the H-ASD + OVA + Tar 5 group were reduced.

It has been reported that aryl hydrocarbon receptor (AhR), which is the receptor for PAHs, activated by highest affinity ligand 2,3,7,8-tetrachlodibenzo-p-dioxin (TCDD) decreased pro-inflammatory cytokines levels and induce the Fox3^+^ Treg cell [24]. LPS-induced production of pro-inflammatory cytokines is augmented in AhR^−/−^ peritoneal macrophages compared with WT cells [25]. Hence, the high dose of Tar might cause the immune suppressive effect by the activation of AhR.

Eosinophilia makes an important contribution to the pathophysiology and pathology of allergic asthma [26]. IL-5 has been recognized as the major maturation, differentiation and recruitment factor for eosinophils [27, 28]. IL-13 is another important factor; in a mouse model of airway effects due to asthma, eosinophilia was markedly inhibited by neutralization of IL-13 [29]. Moreover, IL-13 is known to induce goblet cell proliferation [30] and is a characteristic feature of allergic asthma [31]. In addition, Eotaxin and MCP-3 were also known to be effective eosinophil chemoattractants [32, 33], while the toxic mediators, such as major basic proteins, reactive oxygen radicals, platelet-activating factors, and leukotrienes released from eosinophils, play an important role in inducing bronchial mucosal injury [34]. Therefore, it is not surprising that the Th2 cytokines together with increased eosinophil-relevant chemokines, including Eotaxin and MCP-3, aggravated eosinophilic lung inflammation in the present study. The major basic protein released from eosinophils is known to activate neutrophils and subsequently cause them to release superoxide [35]. On the other hand, the increases of RANTES and MCP-1 may also contribute to the neutrophilia and exacerbation of airway inflammation [36, 37], so it is possible that the airway injury observed in the present study may be the result of the toxic mediators released from eosinophils and neutrophils.

A case control study has shown that exposure to PAHs originating from combustion of organic matters, especially phenanthrene, is associated with childhood asthma and oxidative stress [38]. Several in vitro studies have shown that PAHs benzo[a]pyrene, 1, 6-BaP-quinone (BPQ), and 3, 6-BPQ in incubation with human basophils enhanced histamine release and IL-4 production in response to crosslinking the high-affinity IgE receptor, Fc epsilon RI, and caused a significant increase of reactive oxygen species (ROS) [39]. β-Napthoflavone, a representative PAH, and the quinone metabolites induced Jun kinase and p38 mitogen-activated protein kinase (MAPK) activities and the generation of activator protein-1 (AP-1) in RAW264.7 macrophage cell lines. Activation of MAPK was dependent on generation of oxidative stress [40]. Pyrene, another representative PAH in diesel exhaust, increased IL-8 promoter activity, mRNA production and protein expression in human epithelium cells. AP-1 and nuclear factor-κB (NF-κB) binding sites in the IL-8 promoter regulated IL-8 production [41]. The high level of oxidative stress caused by PAHs activated the NF-κB and MAPKs signaling cascades, which are important for the expression of many genes that participate in pulmonary inflammation [42]. These genes include IL-4, IL-5, IL-10, IL-13, IL-8, RANTES, MIP-1α, MCP-3, GM-CSF, TNF-α, ICAM-1 and VCAM-1 [43]. It is interesting that Tars 1 and 5, in particular Tar 1, markedly enhanced Th2 responses in the presence of OVA and H-ASD, although Tar showed weak Th2 response in the presence of OVA. It is clear that H-ASD has potential to elicit a weak reaction by Tar and OVA. Three mixtures increased IL-4, IL-5, IL-13, RANTES, KC, MIP-1α, and MCP-3. H-ASD might help generation of ROS by PAHs, which present in the Tar used in the present study (Table 2). Therefore, we speculate that the aggravating effect is due to the enhancement of inflammatory mediators via oxidative stress caused by the combination of H-ASD and PAHs in Tar.

On the other hand, in the presence of H-ASD and OVA, Tars 1 and 5 caused the production of OVA-specific IgG1 in serum. It seems that an increase of IgG1 is being interlocked with activation of Th2 response caused by these mixtures. Diesel samples with different levels of PAHs aggravated OVA-induced airway inflammation along with elevated OVA-specific IgG1 [19]. Antigen-specific IgG1 can cause degranulation via an Fc_γ_ RII receptor on the eosinophil’s surface [44]. Therefore, antibodies may play an important role in the aggravation of lung eosinophilia caused by H-ASD + OVA + Tar.

## Conclusions

This study demonstrates that H-ASD with low levels of Tar exacerbates OVA-induced lung eosinophilia via increases in Th2-mediated cytokines and antigen-specific immunoglobulin. The aggravation of the allergic lung inflammation by Tar might be caused by PAHs. Future studies should investigate the mechanism of how ASD-bound PAHs induce their aggravating effects. The results of the current study suggest that exposure to ASD with Tar is a significant risk factor for adult and childhood asthma. Atmospheric exposure to PAHs originating from the combustion of organic matters and desert-dust may influence human respiratory health on a world-wide scale.

## Abbreviations

OVA: Ovalbumin
ASD: Asian sand dust
BALF: Bronchoalveolar lavage fluid
PAHs: Polycyclic aromatic hydrocarbons
ELISA: Enzyme-linked immunosorbent assays
EMSA: Electrophoretic mobility shift assay
H-ASD: Heated Asian sand dust
IFN-γ: Interferon-γ
IL: Interleukin
KC: Keratinocyte chemoattractant
MCP-1: Monocyte chemotactic protein-1
MCP-3: Monocyte chemotactic protein-3
MIP-1α: Macrophage inflammatory protein-1α
RANTES: Regulated on activation normal T cell expressed and presumably secreted
TGF-β: Transforming growth factor-β.

## References

[1] Schwartz J, Slater D, Larson TV, Pierson WE, Koenig JQ (1993). Particulate air pollution and hospital emergency room visits for asthma in Seattle. Am Rev Respir Dis.

[2] Romieu I, Meneses F, Ruiz S, Sienra JJ, Huerta J, White MC, Etzel RA (1996). Effects of air pollution on the respiratory health of asthmatic children living in Mexico City. Am J Respir Crit Care Med.

[3] Cadelis G, Tourres R, Molinie J (2014). Short-term effects of the particulate pollutants contained in saharan dust on the visits of children to the emergency department due to asthmatic conditions in Guadeloupe (French Archipelago of the Caribbean). PLoS One.

[4] Mallone S, Stafoggia M, Faustini A, Gobbi GP, Marconi A, Forastiere F (2011). Saharan dust and associations between particulate matter and daily mortality in Rome, Italy. Environ Health Perspect.

[5] Rutherford S, Clark E, McTainsh G, Simpson R, Mitchell C (1999). Characteristics of rural dust events shown to impact on asthma severity in Brisbane, Australia. Int J Biometeorol.

[6] Yoo Y, Choung JT, Yu J, Kim do K, Koh YY (2008). Acute effects of Asian dust events on respiratory symptoms and peak expiratory flow in children with mild asthma. J Korean Med Sci.

[7] Duce RA, Unni CK, Ray BJ, Prospero JM, Merrill JT (1980). Long-range atmospheric transport of soil dust from Asia to the tropical north pacific: temporal variability. Science.

[8] Husar RB, Tratt DB, Schichtel BA, Falke SR, Li F, Jaffe D, Gasso’ S, Gill T, Laulainen NS, Lu F, Reheis MC, Chun Y, Westphal D, Holben BN, Gueymard C, McKendry I, Kuring N, Feldman GC, McClain C, Frouin RJ, Merrill J, DuBois D, Vignola F, Murayama T, Nickovic S, Wilson WE, Sassen K, Sugimoto N, Malm WC (2001). Asian dust events of April 1998. J Geophys Res.

[9] Kim BG, Han JS, Park SU (2001). Transport SO_2_ and aerosol over the Yellow Sea. Atmos Environ.

[10] Watanabe M, Yamasaki A, Burioka N, Kurai J, Yoneda K, Yoshida A, Igishi T, Fukuoka Y, Nakamoto M, Takeuchi H, Suyama H, Tatsukawa T, Chikumi H, Matsumoto S, Sako T, Hasegawa Y, Okazaki R, Horasaki K, Shimizu E (2011). Correlation between Asian dust storms and worsening asthma in Western Japan. Allergol Int.

[11] Kanatani T, Ito I, Al-Delaimy WK, Adachi Y, Mathews WC, Ramsdell JW (2010). Toyama Asian Desert Dust and Asthma Study Team. Desert dust exposure is associated with increased risk of asthma hospitalization in children. Am J Respir Crit Care Med.

[12] Ichinose T, Yoshida S, Hiyoshi K, Sadakane K, Takano H, Nishikawa M, Mori I, Yanagisawa R, Kawazato H, Shibamoto YA (2008). The effects of microbial materials adhered to Asian sand dust on allergic lung inflammation. Arch Environ Contam Toxicol.

[13] He M, Ichinose T, Yoshida S, Nishikawa M, Mori I, Yanagisawa R, Takano H, Inoue K, Sun G, Shibamoto T (2010). Airborne Asian sand dust enhances murine lung eosinophilia. Inhal Toxicol.

[14] Ren Y, Ichinose T, He M, Song Y, Yoshida Y, Youshida S, Nishikawa M, Takano H, Sun G, Shibamoto T (2014). Enhancement of OVA-induced murine lung eosinophilia by co-exposure to contamination levels of LPS in Asian sand dust and heated dust. Allergy, Asthma Clinical Immunology.

[15] Kim W, Doh SJ, Yu Y, Lee M (2008). Role of Chinese wind-blown dust in enhancing environmental pollution in Metropolitan Seoul. Environ Pollut.

[16] Primbs T, Simonich S, Schmedding D, Wilson G, Jaffe D, Takami A, Kato S, Hatakeyama S, Kajii Y (2007). Atmospheric outflow of anthropogenic semivolatile organic compounds from East Asia in spring 2004. Environ Sci Technol.

[17] Arashidani K, Fukunaga M, Yoshikawa M, Kodama Y, Mizuguchi Y (1982). Mutagenic activities of Benzene extracted of airborne particulates. J Univ Occup Environ Health.

[18] Yanagisawa R, Takano H, Inoue KI, Ichinose T, Sadakane K, Yoshino S, Yamaki K, Yoshikawa T, Hayakawa K (2006). Components of diesel exhaust particles differentially affect Th1/Th2 response in a murine model of allergic airway inflammation. Clin Exp Allergy.

[19] Stevens T, Cho SH, Linak WP, Gilmour MI (2009). Differential potentiation of allergic lung disease in mice exposed to chemically distinct diesel samples. Toxicol Sci.

[20] Nishikawa M, Dashdondog Batdor D, Ukachi M, Onishi K, Nagan K, Mori I, Matsui I, Sano T (2013). Preparation and chemical characterisation of an Asian mineral dust certified reference material. Anal Methods.

[21] Kodama Y, Arashidani K (1983). Simplified analysis of benzo(a)pyrene in airborne particulates by High-performance liquid chromatography. J Chromatogr.

[22] He M, Ichinose T, Song Y, Yoshida Y, Arashidani K, Yoshida S, Liu B, Nishikawa M, Takano H, Sun G (2013). Effects of two Asian sand dusts transported from the dust source regions of Inner Mongolia and northeast China on murine lung eosinophilia. Toxicol Appl Pharmacol.

[23] He M, Ichinose T, Yoshida S, Takano H, Nishikawa M, Sun G, Shibamoto T (2013). Induction of immune tolerance and reduction of aggravated lung eosinophilia by co-exposure to Asian sand dust and ovalbumin for 14 weeks in mice. Allerg Asthma Clin Immunol.

[24] Benson JM, Shepherd DM (2011). Aryl hydrocarbon receptor activation by TCDD reduces inflammation associated with Crohn’s disease. Toxicol Sci.

[25] Kimura A, Naka T, Nakahama T, Chinen I, Masuda K, Nohara K, Fujii-Kuriyama Y, Kishimoto T (2009). Aryl hydrocarbon receptor in combination with Stat1 regulates LPS-induced inflammatory responses. J Exp Med.

[26] Shen HH (2005). Eosinophil: central mediator of allergic asthma?. Chin Med J (Engl).

[27] Rothenberg ME, Hogan SP (2006). The eosinophil. Annu Rev Immunol.

[28] Takatsu K, Nakajima H (2008). IL-5 and eosinophilia. Curr Opin Immunol.

[29] Grünig G, Warnock M, Wakil AE, Venkayya R, Brombacher F, Rennick DM, Sheppard D, Mohrs M, Donaldson DD, Locksley RM, Corry DB (1998). Requirement for IL-13 independently of IL-4 in experimental asthma. Science.

[30] Zhu Z, Homer RJ, Wang Z, Chen Q, Geba GP, Wang J, Zhang Y, Elias JA (1999). Pulmonary expression of interleukin-13 causes inflammation, mucus hypersecretion, subepithelial fibrosis, physiologic abnormalities, and eotaxin production. J Clin Invest.

[31] Shimura S, Andoh Y, Haraguchi M, Shirato K (1996). Continuity of airway goblet cells and intraluminal mucus in the airways of patients with bronchial asthma. Eur Respir J.

[32] Ponath PD, Qin S, Ringler DJ, Clark-Lewis I, Wang J, Kassam N, Smith H, Shi X, Gonzalo JA, Newman W, Gutierrez-Ramos JC, Mackay CR (1996). Cloning of the human eosinophil chemoattractant, eotaxin. Expression, receptor binding, and functional properties suggest a mechanism for the selective recruitment of eosinophils. J Clin Invest.

[33] Dahinden CA, Geiser T, Brunner T, von Tscharner V, Caput D, Ferrara P, Minty A, Baggiolini M (1994). Monocyte chemotactic protein 3 is a most effective basophil- and eosinophil-activating chemokine. J Exp Med.

[34] Gleich GJ (1990). The eosinophil and bronchial asthma: current understanding. J Allergy Clin Immunol.

[35] Haskell MD, Moy JN, Gleich GJ, Thomas LL (1995). Analysis of signaling events associated with activation of neutrophil superoxide anion production by eosinophil granule major basic protein. Blood.

[36] Pan ZZ, Parkyn L, Ray A, Ray P (2000). Inducible lung-specific expression of RANTES: preferential recruitment of neutrophils. Am J Physiol Lung Cell Mol Physiol.

[37] Johnston B, Burns AR, Suematsu M, Issekutz TB, Woodman RC, Kubes P (1999). Chronic inflammation upregulates chemokine receptors and induces neutrophil migration to monocyte chemoattractant protein-1. J Clin Invest.

[38] Suresh R, Shally A, Mahdi AA, Patel DK, Singh VK, Rita M (2009). Assessment of association of polycyclic aromatic hydrocarbons with bronchial asthma and oxidative stress in children: a case–control study. Indian J Occup Environ Med.

[39] Kepley CL, Lauer FT, Oliver JM, Burchiel SW (2003). Environmental polycyclic aromatic hydrocarbons, benzo(a) pyrene (BaP) and BaP-quinones, enhance IgE-mediated histamine release and IL-4 production in human basophils. Clin Immunol.

[40] Ng D, Kokot N, Hiura T, Faris M, Saxon A, Nel A (1998). Macrophage activation by polycyclic aromatic hydrocarbons: evidence for the involvement of stress-activated protein kinases, activator protein-1, and antioxidant response elements. J Immunol.

[41] Bömmel H, Haake M, Luft P, Horejs-Hoeck J, Hein H, Bartels J, Schauer C, Pöschl U, Kracht M, Duschl A (2003). The diesel exhaust component pyrene induces expression of IL-8 but not of eotaxin. Int Immunopharmacol.

[42] Li N, Hao M, Phalen RF, Hinds WC, Nel AE (2003). Particulate air pollutants and asthma. A paradigm for the role of oxidative stress in PM-induced adverse health effects. Clin Immunol.

[43] Pandya RJ, Solomon G, Kinner A, Balmes JR (2002). Diesel exhaust and asthma: hypotheses and molecular mechanisms of action. Environ Health Perspect.

[44] Kaneko M, Swanson MC, Gleich GJ, Kita H (1995). Allergen-specific IgG1 and IgG3 through Fc gamma RII induce eosinophil degranulation. J Clin Invest.

